# *C. elegans* as an *in vivo* model system for the phenotypic drug discovery for treating paraquat poisoning

**DOI:** 10.7717/peerj.12866

**Published:** 2022-02-01

**Authors:** Peng Ji, Hongyuan Li, Yushan Jin, Yinghua Peng, Lihui Zhao, Xiaohui Wang

**Affiliations:** 1 College of Life Science and Technology, Changchun University of Science and Technology, Changchun, China; 2 Laboratory of Chemical Biology, Changchun Institute of Applied Chemistry (CIAC), Chinese Academy of Sciences (CAS), Changchun, China; 3 Department of Immunology and Department of Cell & Systems Biology, University of Toronto, Toronto, Canada; 4 Key Laboratory of Special Animal Molecular Biology of Jilin Province, Specialty Research Institute of Chinese Academy of Agricultural Sciences, Changchun, China; 5 Department of Applied Chemistry and Engineering, University of Science and Technology of China, Hefei, China; 6 Beijing National Laboratory for Molecular Sciences, Beijing, China

**Keywords:** Paraquat, *C. elegans*, Phenotypic drug discovery, Coenzyme Q10

## Abstract

**Background:**

Paraquat (PQ) is an effective and widely used herbicide and causes numerous fatalities by accidental or voluntary ingestion. However, neither the final cytotoxic mechanism nor effective treatments for PQ poisoning have been discovered. Phenotypic drug discovery (PDD), which does not rely on the molecular mechanism of the diseases, is having a renaissance in recent years owing to its potential to address the incompletely understood complexity of diseases. Herein, the *C. elegans* PDD model was established to pave the way for the future phenotypic discovery of potential agents for treating PQ poisoning.

**Methods:**

*C. elegans* were treated with PQ-containing solid medium followed by statistical analysis of worm survival, pharyngeal pumping, and movement ability. Furthermore, coenzyme Q10 (CoQ10) was used to test the *C. elegans* model of PQ poisoning by measuring the levels of reactive oxygen species (ROS) and malondialdehyde (MDA), mitochondrial morphology, and worm survival rate. Additionally, we used the classic mice model of PQ intoxication to evaluate the validity of the *C. elegans* model of PQ poisoning by measuring the effect of CoQ10 as a potential antidote for PQ poisoning.

**Results:**

In the *C. elegans* model of PQ poisoning, 5 mg/mL PQ increased the levels of ROS, MDA content, mitochondrial fragments, which significantly shortened the lifespan, while CoQ10 alleviated these phenotypes. In the mice model of PQ poisoning, CoQ10 increased the chance of survival in PQ poisoned mice while reducing ROS, MDA content in lung tissue and inhibiting PQ-induced lung edema. Moreover, CoQ10 alleviated the lung morphopathological changes induced by PQ.

**Conclusion:**

Here we established a *C. elegans* model of PQ poisoning, whose validity was confirmed by the classic mice model of PQ intoxication.

## Introduction

Paraquat (PQ) is known as one of the most broadly used herbicides in the world (Diaz Kirmser et al., 2010). The high toxicity of PQ, together with its widespread use and ready accessibility, results in thousands of deaths each year by both accidental and deliberate self-poisoning (Dinis-Oliveira et al., 2008), among which intentional suicide deaths currently predominate, especially in developing countries. The lung is the main target organ of PQ intoxication due to its active polyamine uptake transport systems, which concentrate PQ rapidly into alveolar epithelial cells (Cappelletti, Maggioni & Maci, 1998; Dunbar et al., 1988). However, the mechanism of PQ-induced lung toxicity has not been fully understood (Dinis-Oliveira et al., 2008). The main potential mechanism is that PQ results in mitochondrial dysfunction and the production of reactive oxygen species (ROS) (Liu et al., 2018; Zhao et al., 2017). PQ acts by redox cycling, which affects the electron transport chain function and increases the production of superoxide in the mitochondria, disrupting the synthesis of pulmonary surfactants and damaging the vital cellular constituents (Tampo, Tsukamoto & Yonaha, 1999). Intensive studies of PQ toxicology and drug discovery for treating PQ poisoning have been performed (Liu et al., 2019; Shen et al., 2017; Zhang et al., 2019). However, no clinically approved antidote for PQ poisoning has been found.

CoQ10 is a lipid-soluble cofactor that acts as an electron-transfer carrier and is naturally synthesized by mammals and plants. CoQ10 links basic aspects of energy metabolism and antioxidant protection. This naturally occurring compound plays a major role in cellular metabolism since it contributes to oxidative phosphorylation by mediating electron transfer between Complexes I/II and Complex III in the mitochondrial inner membrane (Mantle & Dybring, 2020). Beyond that, CoQ10 has fundamental properties that make it an antioxidant or free radical scavenger and confer its potential benefit in a variety of clinical situations (Mantle & Dybring, 2020; Rabanal-Ruiz, Llanos-Gonzalez & Alcain, 2021).

Target-based drug discovery has been the main method of choice in both academic translational research centers and the pharmaceutical industry in the past two decades (Sams-Dodd, 2007). However, phenotypic drug discovery (PDD), which does not rely on the identity of a specific drug target or a hypothesis about its role in disease, is having a renaissance in recent years owing to its potential to address the incompletely understood complexity of diseases and the promise of delivering first-in-class drugs (Moffat et al., 2017; Zheng, Thorne & McKew, 2013). The mechanisms of PQ toxicity are complicated as all may occur and are likely to be synergistic (Gawarammana & Buckley, 2011; Suntres, 2002). Therefore, PDD would be a more suitable strategy for the discovery of antidotes for PQ poisoning. For more than half a century, *C. elegans* has been extensively used as a genetic model organism (Honnen, 2017). *C. elegans* has emerged as an intact *in vivo* system for PDD in the past decade (Carretero, Solis & Petrascheck, 2017), owing to conservation of cellular processes across species, small size (∼1 mm in length), rapid replication cycle (∼3 days) and ability to produce ∼300 offspring in ∼3 days, low husbandry costs, ease of growing and maintenance as well as manipulation, simple screening assays, and amenability to high-throughput and high content screening methodology (Leung et al., 2013; Maurer et al., 2015; Rangaraju, Solis & Petrascheck, 2015). Herein we aimed to establish a *C. elegans* model of PQ poisoning. Additionally, the classic mice model of PQ intoxication was used to evaluate the validity of *C. elegans* model of PQ poisoning by measuring the effect of CoQ10 as a potential antidote for PQ poisoning.

## Materials & Methods

### Worm culture

The worms were grown on solid nematode growth medium (NGM) plates (3 mg/mL NaCl, 2.5 mg/mL peptone, 20 mg/mL agar, 1 mM MgSO_4_, 50 mg/L cholesterol, 1 mM CaCl_2_, 24.38 mM potassium phosphate buffer (pH 6.0)) in 20 °C incubator according to a previous study (Sutphin & Kaeberlein, 2009). The following strains were used in this work: Bristol strain N2 was used as the wild-type strain, and the transgenic strain was SJ4103 [zcIs14 (*myo-3*::GFP^mit^)]. Nematodes were procured from Caenorhabditis Genetics Center (University of Minnesota, Minnesota). Following the standard protocol, *C. elegans* were fed by the lawn of *Escherichia coli* strain OP50 propagated in LB broth (10 mg/mL tryptone, 5 mg/mL yeast extract, 10 mg/mL NaCl, 10 mM Tris (pH 8.0)) (Sutphin & Kaeberlein, 2009).

### Synchronization of worms

To synchronize adult worms, day 1 post adult worms were allowed to lay eggs for 4–6 h on bacteria-seeded NGM plates and were subsequently removed from the plates. The remaining eggs were cultured to obtain a synchronous population.

### Establishing the *C. elegans* model of PQ poisoning

Synchronized wild-type *C. elegans* (N2) at the L4 stage were grown on floxuridine (FUDR)-added NGM/OP50 plates for 24 h. Worms were then transferred to PQ (0, 5, 10, 20 mg/mL)-containing NGM/OP50 plates for 3 h. Worms were next transferred to new NGM/OP50 plates containing FUDR. The mortality rate of worms, locomotion behavior, and pharyngeal pumping were determined immediately. Locomotion behavior was quantified by monitoring body thrashing for 1 min. A movement of the worm that swings its head and/or tail to the same side was counted as one thrash. The pharyngeal pumping assay was evaluated by measuring the number of pharyngeal contractions in 30 s. The survival of the nematodes transferred to new NGM/OP50 plates after exposure to paraquat (5 mg/mL) was observed, which was checked every day with a soft touch at the tip of the pharynx of the worms. Each assay was repeated three times.

### *C. elegans* lifespan measurement

Synchronized N2 worms at the L4 stage were plated on FUDR-added NGM/OP50 plates for 24 h. Worms were then transferred to PQ (5 mg/mL)-containing solid plates for 3 h. Worms were next transferred to FUDR added solid plates containing various concentrations of coenzyme Q10 (CoQ10, Fig. S1) (Energy Chemical, Shanghai, China) or without CoQ10 and incubated at 20 °C. The worm population was transferred every day during the lifespan assay, and animals were scored for survival. CoQ10 was dissolved in Tween 80 (0.1%) + glycerol (0.1%) solution. The control population was treated with Tween 80 (0.1%) + glycerol (0.1%) solution

### *C. elegans* ROS assay

H_2_DCF-DA (Sigma–Aldrich, St. Louis, MO, USA) was used as the fluorescence probe to measure ROS in *C. elegans* based on the formation of highly fluorescent 2′, 7′-dichlorofluorescein (DCF) from nonfluorescent H_2_DCF-DA by reacting with ROS. Synchronized N2 worms at the L4 stage were plated on FUDR-added NGM/OP50 plates for 24 h. Worms were then transferred to PQ (5 mg/mL)-containing solid plates for 3 h, and next transferred to FUDR added solid plates containing 1.8 mg/mL CoQ10 or without CoQ10 and incubated at 20 °C. On Day 5, ROS levels were measured by H_2_DCF-DA staining. For imaging detection, the worms were washed with M9 buffer 3 times and then stained with 50 µM H_2_DCF-DA for 1 h. After three washes with M9 buffer, worms were imaged with a Nikon TS2-FL fluorescence microscope. The fluorescence intensity of the images was quantified using ImageJ software (http://rsb.info.nih.gov/ij/). The values in the experimental groups were normalized to the control group and subjected to analysis of relative fluorescence intensity. For each condition, up to 30 worms were observed and imaged. The assay was repeated in three independent experiments.

### *C. elegans* mitochondrial imaging

*C. elegans* strain SJ4103 expressing mitochondrial-targeted green fluorescent protein (GFP) driven by the muscle-specific *myo-3* promoter was used for mitochondrial imaging. Quantitative analysis of mitochondrial morphology in worms was performed by measuring mitochondrial area. Synchronized SJ4103 worms at the L4 stage were plated on FUDR-added NGM/OP50 plates for 24 h. Worms were then transferred to PQ (5 mg/mL)-containing solid plates for 3 h, and next transferred to FUDR added solid plates containing 1.8 mg/mL CoQ10 or without CoQ10 and incubated at 20 °C. On day 5, worms were immobilized with levamisole before mounting on 2% agarose pads for microscopic examination with a Nikon TS2-FL fluorescence microscope. All snapshots were taken from the same part of *C. elegans*: muscle cells from the upper part of the worm. Mitochondria were segmented from the background by setting the pixel intensity threshold. The mitochondrial area was measured using ImageJ software (http://rsb.info.nih.gov/ij/). For each condition, up to 15 worms were observed and imaged. The values in the experimental groups were normalized to the control group. This experiment was repeated three times.

### *C. elegans* malondialdehyde (MDA) measurements

Synchronized N2 worms at the L4 stage were plated on FUDR-added NGM/OP50 plates for 24 h. Worms were then transferred to PQ (5 mg/mL)-containing solid plates for 3 h, and next transferred to FUDR added solid plates containing 1.8 mg/mL CoQ10 or without CoQ10 and incubated at 20 °C. On day 5, the pretreated worms were sonicated using an Ultrasonics Processor KBS-900 (Kunshan Ultrasonic Instrument Co., Shanghai, China) with 2-sec pulse on and 4-sec pulse off at 4 °C for 2 min. The MDA (Fig. S1) content was measured by assay kits (Leagene Biotechnology, Beijing, China) according to the manufacturer’s instructions. The MDA content was normalized to the protein concentration. The values in the experimental groups were normalized to the control group to analyze the relative MDA content. This experiment was repeated three times.

### *In vivo* mice study

All the mice used in our experiments were male. SPF C57BL/6 mice (6–7 weeks old) were obtained from Liaoning Changsheng Biotechnology Co., Ltd. The mice were raised at 22−24 °C with relative humidity of 50–70% under 12 h light-dark illumination. The animals were housed in plastic cages (maximum 5 per cage). Standard diet and drinking water for raising mice were used to feed the mice. All procedures treated with mice were in compliance with the Regulations for the Administration of Affairs Concerning Experimental Animals in China. Protocols were approved by the Institutional Animal Care and Use Committee (IACUC) of Changchun Institute of Applied Chemistry, Chinese Academy of Sciences (CIAC2020-84). Note that the study was exploratory and not preregistered. Neither randomization nor blinding methods were used for the selection of animals. The sample sizes were estimated based on prior experience with variability and requirements to identify significant differences. In all studies, no mice were excluded from analyses.

Animals were observed daily throughout the course of the experiment and defined criteria for premature euthanasia (loss of body weight >20%, loss of ability to ambulate (inability to access food or water), unconsciousness with no response to external stimuli) were closely followed. Adequate measures were taken to minimize the pain of experimental animals. After the experiment, the surviving mice were euthanized with isoflurane.

### Survival assay

In total 40 mice were randomly divided into four groups (*n* = 10/each): Control, PQ (intragastrically (*i.g.*) administered with 80 mg/kg); PQ (*i.g.* 80 mg/kg) + CoQ10 (*i.g.* 62.5 mg/kg 1 h after PQ dosing); PQ (*i.g.* 80 mg/kg) + CoQ10 (*i.g.* 125 mg/kg 1 h after PQ dosing). The animals were monitored every 24 h from the onset of PQ exposure for 7 days and the survival rate was recorded.

### PQ-induced lung injury analysis

In total, 24 mice were randomly divided into groups (*n* = 6/each) and administered with PQ (80 mg/kg) and CoQ10 (0, 62.5 and 125 mg/kg) as described in the above survival assay. After 48 h of PQ exposure, surviving mice were anesthetized, and lung lobe samples of mice were collected surgically. The left lung lobe tissues were homogenized, and the supernatants were collected for measuring MDA. The right lower lung lobes were homogenized, and the supernatants were collected for measuring ROS. According to the manufacturer’s instructions, MDA and ROS measurements were performed by assay kits (Nanjing JianCheng Bioengineering Institute, Nanjing, China). The right middle lung lobe of mice was weighed and dried for 48 h in a constant temperature oven at 70 °C, and the dry weight was obtained. Lung wet/dry weight ratio=lung wet weight/lung dry weight ×100%. The values in the experimental groups were normalized to their respective control group and subjected to analysis.

Using standard procedures, right upper lung lobe fixation and HE staining were carried out as described (Chen et al., 2013). Slides (*n* = 3) were scanned at 400 × magnifications. At least two nonconsecutive slides per block were used to analyze whether inflammation was present.

### Statistical analysis

The data were analyzed using GraphPad Prism 5 software (GraphPad Software, La Jolla, CA, USA). The comparisons of the mean values of the analyzed parameters were performed using one-way ANOVA. Survival analyses were performed using the Kaplan–Meier method, and the significance of differences between survival curves was calculated using the log-rank test. For all experiments, significance was accepted at *p* < 0.05.

## Results

### Establishing *C. elegans* as a model for PQ poisoning

**Figure 1:**
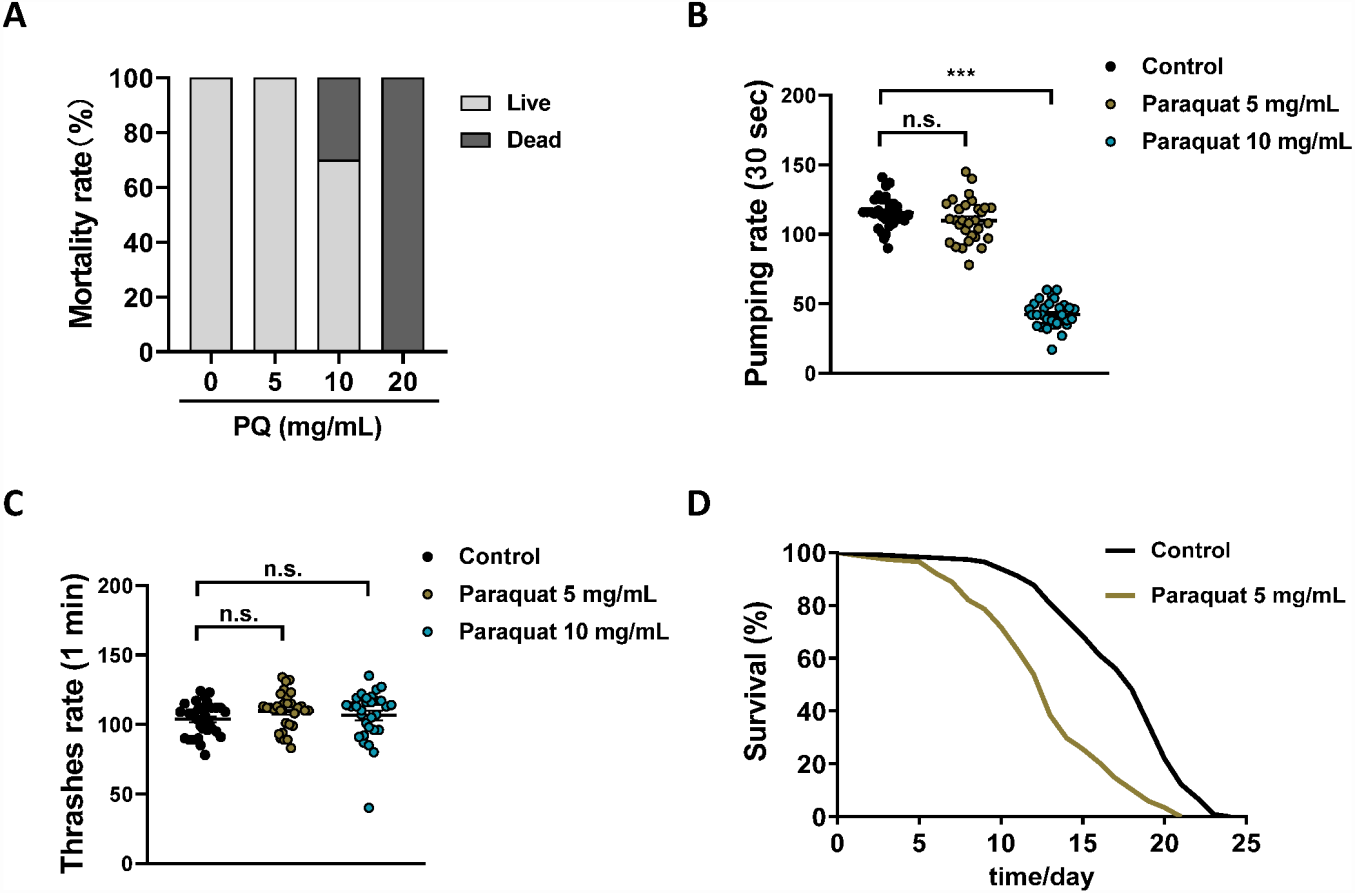
Establishing the *C. elegans* as a model for PQ poisoning. Synchronized N2 nematode populations were collected from the NGM plates at the day 1 of adulthood, subsequently exposed to different concentrations of PQ (0, 5, 10, 20 mg/mL) for 3 h. Mortality (A, *n* = 120), pharyngeal pumping (B, *n* = 30) and body bending (C, *n* = 30) were measured. (D) Survival curve of N2 worms after PQ (5 mg/mL) poisoning. Control, *n* = 114; Paraquat 5 mg/mL, *n* = 117. Data are representative of three independent experiments. ^∗∗∗^*p* < 0.001; *n.s.* , non-significant.

Despite intensive studies of PQ toxicity, neither the final cytotoxic mechanism nor a clinically useful antidote has been discovered (Dinis-Oliveira et al., 2008). To find potential therapeutics for treating PQ poisoning, an animal model suitable for drug screening is urgently needed. *C. elegans* is an excellent *in vivo* system for PDD (Carretero, Solis & Petrascheck, 2017). Herein, we tried to establish a *C. elegans* model of PQ poisoning. To mimic human PQ poisoning, N2 wild-type worms were exposed to different concentrations (0, 5, 10, 20 mg/mL) of PQ for 3 h, owing to the poor permeability of the cuticle (Van de Walle et al., 2019). It should be noted that when the PQ treatment times were less than 3 h, the experimental variability was large. As shown in Fig. 1A, 20 mg/mL PQ killed all the worms immediately after 3 h of treatment, 10 mg/mL PQ killed 30% of the worms, while 5 mg/mL PQ did not affect the survival of the worms. Meanwhile, the influence of PQ treatment on the physiology of N2 wild-type worms was further checked. PQ (10 mg/mL) severely affected the pumping rate of worms, while 5 mg/mL PQ treatment had no apparent effect on the pumping rate (Fig. 1B). PQ (5 and 10 mg/mL) did not affect body bends after 3 h of treatment (Fig. 1C). Unlike organic phosphorus pesticides, which are acetylcholinesterases with rapid onset of immediate symptoms following ingestion (Soares et al., 2019), ingestion of a moderate dose of PQ usually produces no symptoms except for possible corrosive lesions during the first phase of PQ poisoning (Dinis-Oliveira et al., 2008). Therefore, 5 mg/mL PQ, which did not significantly affect the physiology of N2 wild-type worms during the process (3 h) of PQ poisoning, was chosen as the working concentration for the *C. elegans* model of PQ poisoning. As shown in Fig. 1D and Table S1, PQ (5 mg/mL) poisoning significantly shortened the lifespan of N2 worms compared to the control group (12.5 ± 0.5 days versus 18.0 ± 0.6 days, *p* < 0.01), which indicated that the *C. elegans* model of PQ poisoning was successfully established.

It should be noted that PQ has been widely used in oxidative stress studies and the PQ-induced Parkinson’s disease model in *C. elegans* (Dilberger et al., 2019; Wu et al., 2018). However, the typical PQ concentrations (0.2–1.6 mM) used in these studies (Wu et al., 2018) were much lower than the PQ (5 mg/mL; *i.e*., ∼20 mM) used herein for the PQ poisoning model. The dose of PQ does matter, and the toxicology of PQ at high doses is quite different from that of the mild dose of PQ (Dinis-Oliveira et al., 2008). This study reports the *C. elegans* model of PQ poisoning, which is obviously different from PQ-induced oxidative stress and Parkinson’s disease studies.

### The evaluation of *C. elegans* model of PQ poisoning

**Figure 2:**
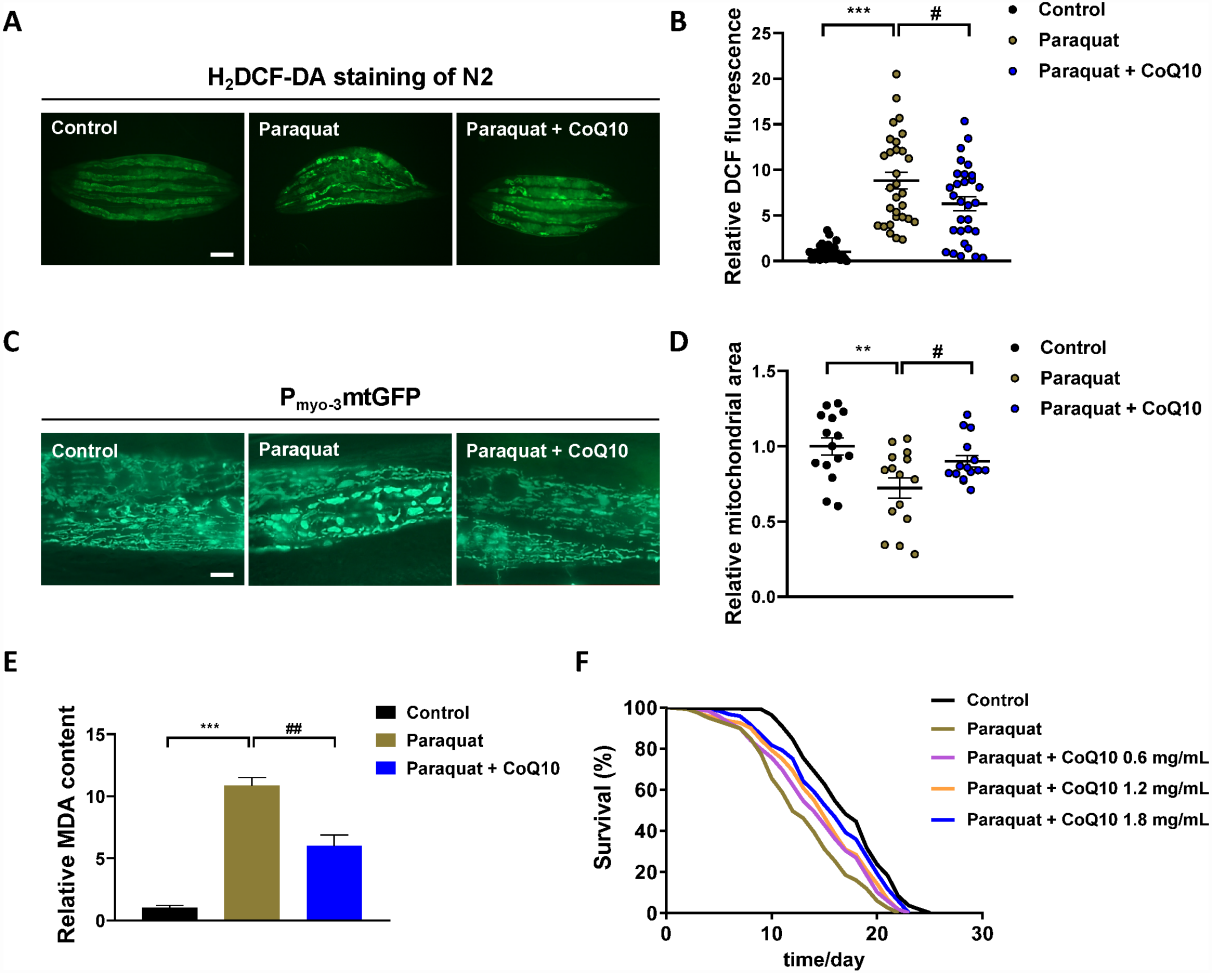
The evaluation of *C. elegans* model of PQ poisoning by CoQ10. One-day-old worms exposed to PQ (5 mg/mL) poisoning for 3 h, and subsequently transferred to new NGM–OP50 plates or NGM–OP50 plates containing 1.8 mg/mL CoQ10. Physiological endpoints were measured on the day 5. (A) Endogenous ROS was measured through end-point microscope DCF fluorescence imaging. Scale bar, 200 µm. (B) Quantified fluorescence intensity of DCF of each group. *n* = 30. (C) Representative pictures of mitochondria in the *C. elegans* transgenic strain SJ4103 carrying a mitochondrial GFP-reporter driven by the muscle-specific *myo-3* promoter. Scale bar, 10 µm. (D) GFP quantification of the mitochondrial area in the muscle of each group. *n* = 15. (E) MDA was detected by assay kit according to the manufacture’s instruction. (F), Survival curves of N2 worms treated with different concentrations of CoQ10 after PQ (5 mg/mL) poisoning. Control, *n* = 110; Paraquat, *n* = 119; Paraquat + CoQ10 (0.6 mg/mL), *n* = 119; Paraquat + CoQ10 (1.2 mg/mL), *n* = 120; Paraquat + CoQ10 (1.8 mg/mL), *n* = 120. Data are representative of three independent experiments. ^∗∗^*p* < 0.01; ^∗∗∗^*p* < 0.001. ^#^*p* < 0.05; ^##^*p* < 0.01.

To evaluate the *C. elegans* model of PQ poisoning, we next tested this *in vivo* model system for PDD. CoQ10 (Fig. S1), which is both an electron transporter in mitochondrial respiratory chain complexes (I, II, and III) and an excellent free radical scavenger (Cui & Kawamukai, 2009), was discovered as a potential therapeutic for neuronal damage induced by PQ (McCarthy et al., 2004). PQ (5 mg/mL) poisoning significantly increased ROS in N2 worms compared to the control worms (Figs. 2A–2B), which is consistent with the observations that PQ exerts its toxic effects primarily through the production of ROS observed in rodents, rabbits and humans (Brigelius et al., 1986; Gómez-Mendikute & Cajaraville, 2003; Mirzaee et al., 2019). CoQ10 (1.8 mg/ml) greatly suppressed PQ-induced ROS in N2 worms (Figs. 2A–2B).

Recent studies have found that the PQ-induced increase in overall cell level ROS originates mainly from mitochondria (Zhao et al., 2017). We investigated the mitochondrial morphology in muscle cells with the transgenic strain SJ4103 [zcIs14 (*myo-3*::GFP^mit^)]. It was observed that PQ increased mitochondrial fragments with the characteristic of increased mitochondrial circularity along with decrease mitochondrial volume (Figs. 2C–2D). CoQ10 was found to reverse the PQ-induced mitochondrial morphology change (Figs. 2C–2D).

MDA is a final product of polyunsaturated fatty acids peroxidation of membrane lipids (Takizawa et al., 2007). An increase in ROS causes the overproduction of MDA (Fig. S1), an indicator of oxidative stress (Gawel et al., 2004). As shown in Fig. 2E, PQ increased the MDA level, and CoQ10 strongly inhibited PQ-induced MDA.

Together, these data showed that CoQ10 protected the oxidative stress and mitochondrial morphology change induced by PQ in *C. elegans*, which implied that CoQ10 might have the chance of treating PQ poisoning. As shown in Fig. 2F and Table S2, worms were transferred to NGM plates with different concentrations (0, 0.6, 1.2 and 1.8 mg/mL) of CoQ10 after PQ (5 mg/mL) intoxication, and CoQ10 was found to attenuate PQ-induced mortality in a dose-dependent manner.

We next examined the effects of CoQ10 on *C. elegans* under normal culture conditions. Relative ROS production in worms treated with CoQ10 (1.8 mg/ml) was significantly enhanced compared with ROS production in untreated populations of worms (*p* < 0.001) (Figs. S2A, S2B). Under treatment with CoQ10 (1.8 mg/ml), intact mitochondria were no longer observed such that all mitochondria displayed a disrupted morphology (Fig. S2C). Furthermore, the relative mitochondrial area was dramatically reduced by 68% in the presence of CoQ10 (1.8 mg/ml) (Fig. S2D). CoQ10 (1.8 mg/ml) caused a significant increase in MDA levels compared to those in untreated worms (*p* < 0.001) (Fig. S2E). The results were unanticipated. As shown in Fig. S2F and Table S3, 0.6 or 1.2 mg/ml CoQ10 significantly extended the lifespan of wild-type worms, while CoQ10 (1.8 mg/ml) decreased the lifespan.

### Mice model of PQ poisoning

**Figure 3:**
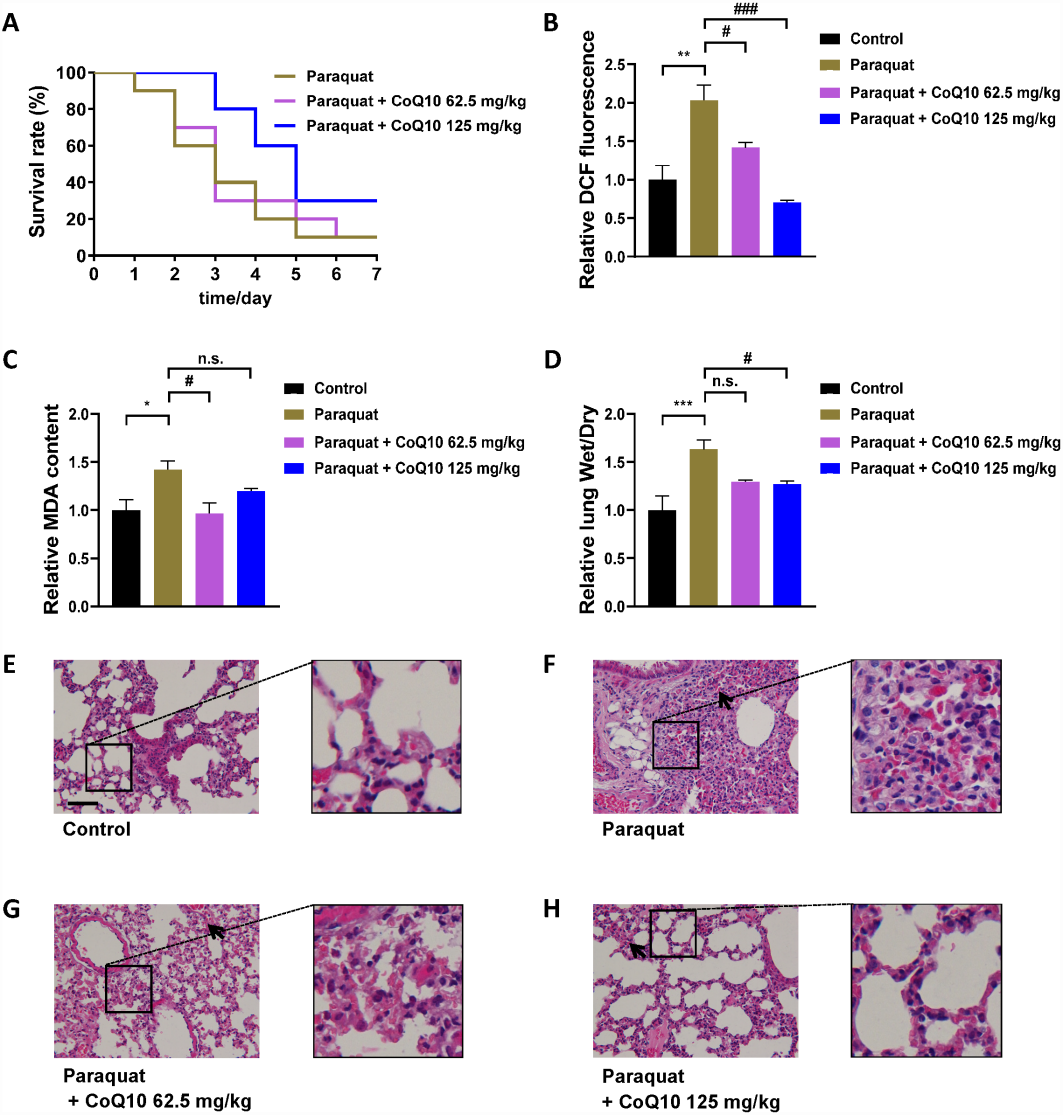
The evaluation of CoQ10 by mice model of PQ poisoning. (A) Survival curves of mice treated with different concentrations of CoQ10 after PQ poisoning (80 mg/kg). (B) Effect of CoQ10 on the PQ-induced lung ROS. (C) Effect of CoQ10 on the PQ-induced MDA in the lung. (D) Effect of CoQ10 on the PQ-induced acute lung injury measured by lung wet/dry weight ratio. (E–H) Morphopathological changes in lung tissues of mice were analyzed by H&E staining. Insets are enlarged regions of corresponding boxes in their respective figures. Scale bar, 100 µm. The black arrows show intraalveolar hemorrhage in lung tissue of mice. ^∗^*p* < 0.05; ^∗∗^*p* < 0.01; ^∗∗∗^*p* < 0.001. ^#^*p* < 0.05; ^###^*p* < 0.001; *n.s.*, non-significant.

To further evaluate the validity of the *C. elegans* model of PQ poisoning, CoQ10, which was discovered as a potential therapeutic agent for PQ poisoning by *C. elegans*, was tested by the classic mice model of PQ poisoning. Given the considerable toxicity of PQ, mice were administered PQ at the dose of 80 mg/kg, which caused a mice mortality rate of approximately 80–90% but not 100%. Different doses of CoQ10 were given 1 h after PQ intoxication. A study in rats has shown that CoQ10 is safe and well tolerated even at high doses (3,000 mg/kg per day) (Wang et al., 2007). The initial dosing regimen of CoQ10 was 62.5 and 125 mg/kg day. As shown in Fig. 3A, PQ exposure was shown to cause rapid progression of death, and a single administration of CoQ10 increased the survival rate of mice with PQ poisoning in a dose-dependent manner. The main molecular mechanism of PQ toxicity is based on its redox cycling and intracellular oxidative stress generation (Clejan & Cederbaum, 1989; Yeh et al., 2006). As expected, PQ-induced a significant increase in ROS (Fig. 3B) and MDA (Fig. 3C) in the lung tissues, which could cause endothelial damage, expansion of vessel permeability, and pulmonary edema (Riahi et al., 2010), while CoQ10 inhibited PQ-induced oxidative stress. As expected, PQ treatment significantly increased the lung wet/dry weight ratio, indicating the presence of pulmonary congestion and edema (Fig. 3D), and CoQ10 inhibited PQ-induced lung edema (Fig. 3D). As shown in Figs. 3E–3H, PQ-induced lung morphopathological changes were assessed by H&E staining. There were diffuse alveolar collapses with thickening of airway smooth muscle, as well as perialveolar, peribronchial, and interstitial fibrosis in lung tissue sections of PQ group mice. These PQ-induced pathological changes were markedly ameliorated by CoQ10. Consistent with the discovery from the *C. elegans* model of PQ poisoning, the mice results demonstrated the role of CoQ10 in blocking PQ-induced oxidative stress, edema, and fibrosis and therefore attenuated PQ-induced death. These *in vivo* data obtained from the mice model support the validity of the *C. elegans* model of PQ poisoning.

## Discussion

After the introduction of PQ, it has been a serious hazard to humans, not with its proper use, but mainly as a result of accidental and voluntary ingestion. For more than half a century after the first reports of PQ poisoning in humans, recovery in such cases remains poor. Since the molecular mechanisms of PQ toxicity are complicated, therapeutics for the treatment of PQ poisoning are virtually nonexistent (Dinis-Oliveira et al., 2008). PDD using an intact *in vivo* system that can closely mimic clinical responses would be more suitable than the target-based strategy for the discovery of antidotes for PQ poisoning (Swinney, 2013). Herein we established a *C. elegans* model of PQ poisoning and confirmed its validity by the classic mice model of PQ poisoning. PQ was found to cause oxidative stress and damage in both *C. elegans* and mice. Considering that *C. elegans* directly contacted PQ while the lung tissue of mice did not, it is not surprising that PQ caused higher ROS (Fig. 2B) and MDA (Fig. 2E) in *C. elegans* than in the lung tissue of mice (Figs. 3B–3C). The large physiological differences between *C. elegans* and mice should be acknowledged. It would be unlikely that *C. elegans* reacts in a quantitatively equivalent way as mouse to PQ treatment.

To evaluate the PDD system of *C. elegans*, CoQ10 was chosen as an agent for treating PQ poisoning in both *C. elegans* and mice. Administration of CoQ10 resulted in a remarkable increase in the median survival time of *C. elegans* after PQ intoxication, which was associated with blockade of PQ-induced oxidative stress and damage. Considering the physiological differences between *C. elegans* and mice, the mice model of PQ poisoning is always necessary to confirm the discovered potential PQ antidote derived from worm-based PDD. The measurements of ROS, MDA and wet/dry weight ratio in the lung, the morphopathological observation of lung tissue,and the survival curves of the mice model notably support the validity of the *C. elegans* model of PQ poisoning. We will screen candidate drugs for PQ detoxification in future experiments, and the application value based on the *C. elegans* model of PQ poisoning is worth further verification in follow-up work.

Currently, there are several clinical trials of CoQ10 in cardiovascular disease, metabolic syndrome, diabetes, kidney disease, neurodegenerative diseases, and male infertility, owing to the antioxidant effect of CoQ10 (Hernández-Camacho et al., 2018). We unexpectedly discovered that high-dose addition of CoQ10 caused oxidative damage to wild-type worms under normal culture conditions and shortened the lifespan of *C. elegans*. This might indicate that the use of CoQ10 within a certain concentration range has the risk of potential oxidative damage. The therapeutic applications of CoQ10 are greatly limited by its poor bioavailability due to its poor solubility in aqueous media and taking too long time to diffuse through cellular membranes (Bhagavan & Chopra, 2006). To overcome this major limitation in PQ poisoning patients requiring emergency treatment, a water soluble CoQ10 formulation, which can be safely administered by intravenous injection, might offer a better treatment effect. Although this study demonstrates that CoQ10 could be therapeutic for detoxification of PQ poisoning, much remains to be done for the convincing efficacy of CoQ10 at the clinical level.

## Conclusions

In conclusion, this study provides a validated *C. elegans* model of PQ poisoning, which paves the way for the phenotypic discovery of potential agents for treating PQ poisoning. It should be acknowledged the physiological differences between C. *elegans* and mice models. The organizational complexity of *C. elegans* is much simpler than that of mammalian animals. It is not surprising that *C. elegans* has greater physiological plasticity, which increases their adaptation to adverse conditions and survival rate compared to higher organisms. Therefore, mammalian animal models of PQ poisoning are always necessary to confirm the discovered potential PQ antidote from the worm model.

##  Supplemental Information

10.7717/peerj.12866/supp-1 Supplemental Information 1 File listing the source data underlying Figs. 1 and 2 analysis Data for the different parts of each of the figures.

10.7717/peerj.12866/supp-2 Supplemental Information 2 File listing the source data underlying Fig. S2 analysis Data for the different parts of each of the figures.

10.7717/peerj.12866/supp-3 Supplemental Information 3 File listing the source data underlying Fig. 3 analysis Data for the different parts of each of the figures.

10.7717/peerj.12866/supp-4 Supplemental Information 4 Effects of PQ (5 mg/mL) on the median lifespan of N2 worms. Related to Fig. 1D SEM: standard error of the mean. The total number of observations equals the number of three independent experiment animals that died plus the number censored. Animals that crawled off the plate, bagged, or burst were censored and therefore excluded from all analysis. *p* values were calculated by utilizing N2 as the control. All statistical analysis was carried out using Graphpad Prism 5 software. The log-rank (Mantel-Cox) test was used for statistical analysis.

10.7717/peerj.12866/supp-5 Supplemental Information 5 Median lifespans of N2 worms after treatment with PQ (5 mg/mL) and different concentrations of CoQ10. Related to Fig. 2F SEM: standard error of the mean. The total number of observations equals the number of three independent experiment animals that died plus the number censored. Animals that crawled off the plate, bagged, or burst were censored and therefore excluded from all analysis. *p* values were calculated as follows: ^*a*^N2, ^*b*^N2-PQ as the control. All statistical analysis was carried out using Graphpad Prism 5 software. The log-rank (Mantel-Cox) test was used for statistical analysis.

10.7717/peerj.12866/supp-6 Supplemental Information 6 Median lifespan of N2 worms exposed to different concentrations of CoQ10. Related to Fig. S2F SD, standard deviation. The total number of observations equals the number of two independent experiment animals that died plus the number censored. Animals that crawled off the plate, bagged, or burst were censored and therefore excluded from all analysis. *p* values were calculated as follows: N2 as the control. All statistical analysis was carried out using Graphpad Prism 5 software. The log-rank (Mantel-Cox) test was used for statistical analysis.

10.7717/peerj.12866/supp-7 Supplemental Information 7 Chemical structures of CoQ10 and MDA

10.7717/peerj.12866/supp-8 Supplemental Information 8 Effects of CoQ10 on ROS, mitochondrial morphology, MDA, and lifespan in *C. elegans* One-day-old worms were transferred to NGM–OP50 plates or NGM–OP50 plates containing 1.8 mg/mL CoQ10. Physiological endpoints were measured on the day 5. (A) Endogenous ROS was measured through end point microscope DCF fluorescence imaging. Scale bar, 200 µm. (B) Quantified fluorescence intensity of DCF in each group. *n* = 29. (C) Representative pictures of mitochondria in the *C.elegans* transgenic strain SJ4103 carrying a mitochondrial GFP reporter driven by the muscle-specific *myo-3* promoter. Scale bar, 10 µm. (D) GFP quantification of the mitochondrial area in the muscle of each group. *n* = 15. (E) MDA was detected by an assay kit according to the manufacturer’s instruction. (F), Survival curves of N2 worms treated with different concentrations of CoQ10. Control, *n* = 63; CoQ10 (0.6 mg/mL), *n* = 66; CoQ10 (1.2 mg/mL), *n* = 58; CoQ10 (1.8 mg/mL), *n* = 67. Each experiment was repeated at least twice. ^∗∗∗^ *p* < 0.001.

10.7717/peerj.12866/supp-9 Supplemental Information 9 The ARRIVE guidelines 2.0: author checklist

## Additional Information and Declarations

### Data Availability

The following information was supplied regarding data availability: The raw measurements are available in the Supplementary Files.
