# Supplementary material for: C. elegans as an in vivo model system for the phenotypic drug discovery for treating paraquat poisoning
Source: PeerJ. 2022 Feb 1;10:e12866. doi: 10.7717/peerj.12866 (PMC8815376; doi:10.7717/peerj.12866)
Supplement: Supplemental Information 4 — SEM: standard error of the mean. The total number of observations equals the number of three independent experiment animals that died plus the number censored. Animals that crawled off the plate, bagged, or burst were censored and therefore excluded from all analysis. p values were calculated by utilizing N2 as the control. All statistical analysis was carried out using Graphpad Prism 5 software. The log-rank (Mantel-Cox) test was used for statistical analysis. [file peerj-10-12866-s004.doc]

**Table S1:**

**Effects of PQ (5 mg/mL) on the median lifespan of N2 worms. Related to Fig. 1D.** SEM: standard error of the mean. The total number of observations equals the number of three independent experiment animals that died plus the number censored. Animals that crawled off the plate, bagged, or burst were censored and therefore excluded from all analysis. *p* values were calculated by utilizing N2 as the control. All statistical analysis was carried out using Graphpad Prism 5 software. The log-rank (Mantel-Cox) test was used for statistical analysis.

|  | **Median lifes**  **± SEM**  **20 ^o^C (Days)** | ***p* Value** | **Total Animals Died/Total** |
| --- | --- | --- | --- |
|  | **Trial # 1** |  |  |
| **N2** | **17.0** | **-** | **39/40** |
| **N2-Paraquat** **(5 mg/mL)** | **12.0** | **< 0.0001** | **40/40** |
|  | **Trial # 2** |  |  |
| **N2** | **18.0** | **-** | **37/40** |
| **N2-Paraquat (5 mg/mL)** | **13.5** | **0.0007** | **38/40** |
|  | **Trial # 3** |  |  |
| **N2** | **19.0** | **-** | **38/40** |
| **N2-Paraquat (5 mg/mL)** | **12.0** | **< 0.0001** | **39/40** |
|  | **Total** |  |  |
|  | **Median lifes**  **± SEM**  **20 ^o^C (Days)** | ***p* Value** | **Total Animals** |
| **N2** | **18.0 ± 0.6** | **-** | **114** |
| **N2-Paraquat (5 mg/mL)** | **12.5 ± 0.5** | **< 0.0001** | **117** |
