# Supplementary material for: C. elegans as an in vivo model system for the phenotypic drug discovery for treating paraquat poisoning
Source: PeerJ. 2022 Feb 1;10:e12866. doi: 10.7717/peerj.12866 (PMC8815376; doi:10.7717/peerj.12866)
Supplement: Supplemental Information 6 — SD, standard deviation. The total number of observations equals the number of two independent experiment animals that died plus the number censored. Animals that crawled off the plate, bagged, or burst were censored and therefore excluded from all analysis. p values were calculated as follows: N2 as the control. All statistical analysis was carried out using Graphpad Prism 5 software. The log-rank (Mantel-Cox) test was used for statistical analysis. [file peerj-10-12866-s006.doc]

**Table S3:**

**Median lifespan of N2 worms exposed to different concentrations of CoQ10. Related to Fig. S2F.** SD: standard deviation. The total number of observations equals the number of two independent experiment animals that died plus the number censored. Animals that crawled off the plate, bagged, or burst were censored and therefore excluded from all analysis. *p* values were calculated as follows: N2 as the control. All statistical analysis was carried out using Graphpad Prism 5 software. The log-rank (Mantel-Cox) test was used for statistical analysis.

|  | **Median lifes**  **± SD**  **20 ^o^C (Days)** | ***p* Value** | **Total Animals Died/Total** |
| --- | --- | --- | --- |
|  | **Trial # 1** |  |  |
| **N2** | **19.5** | **-** | **34/34** |
| **N2-CoQ10 (0.6 mg/mL)** | **27.5** | **< 0.0001** | **34/34** |
| **N2-CoQ10 (1.2 mg/mL)** | **22.0** | **0.0211** | **29/29** |
| **N2-CoQ10 (1.8 mg/mL)** | **16.0** | **0.1552** | **31/31** |
|  | **Trial # 2** |  |  |
| **N2** | **19** | **-** | **29/29** |
| **N2-CoQ10 (0.6 mg/mL)** | **28.0** | **< 0.0001** | **32/32** |
| **N2-CoQ10 (1.2 mg/mL)** | **21.0** | **0.1387** | **29/29** |
| **N2-CoQ10 (1.8 mg/mL)** | **16.5** | **0.0678** | **36/36** |
|  | **Total** |  |  |
|  | **Median lifes**  **± SD**  **20 ^o^C (Days)** | ***p* Value** | **Total Animals** |
| **N2** | **19.3 ± 0.4** | **-** | **63** |
| **N2-CoQ10 (0.6 mg/mL)** | **27.8 ± 0.4** | **< 0.0001** | **66** |
| **N2-CoQ10 (1.2 mg/mL)** | **21.5 ± 0.7** | **0.008** | **58** |
| **N2-CoQ10 (1.8 mg/mL)** | **16.3 ± 0.4** | **< 0.0258** | **67** |
