# Supplementary figures and images for: C. elegans as an in vivo model system for the phenotypic drug discovery for treating paraquat poisoning
Source: PeerJ. 2022 Feb 1;10:e12866. doi: 10.7717/peerj.12866 (PMC8815376; doi:10.7717/peerj.12866)

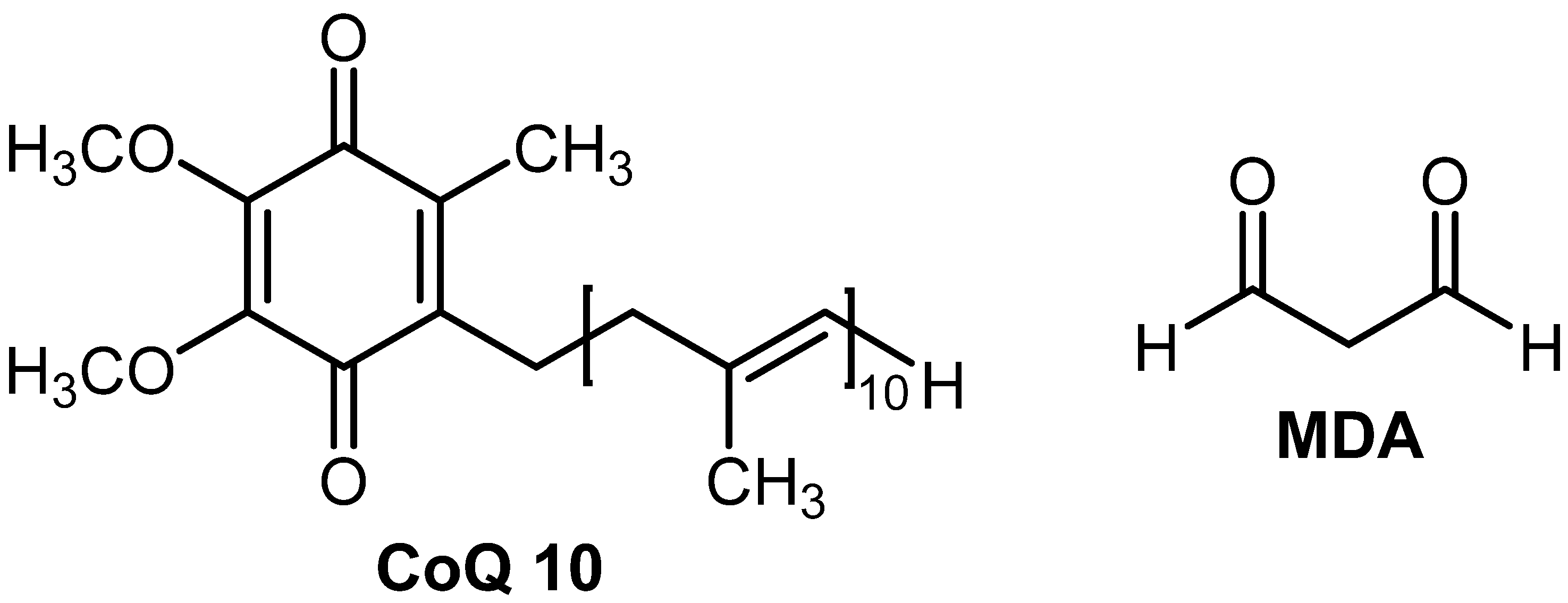

Supplement: Supplemental Information 7 [file peerj-10-12866-s007.png]

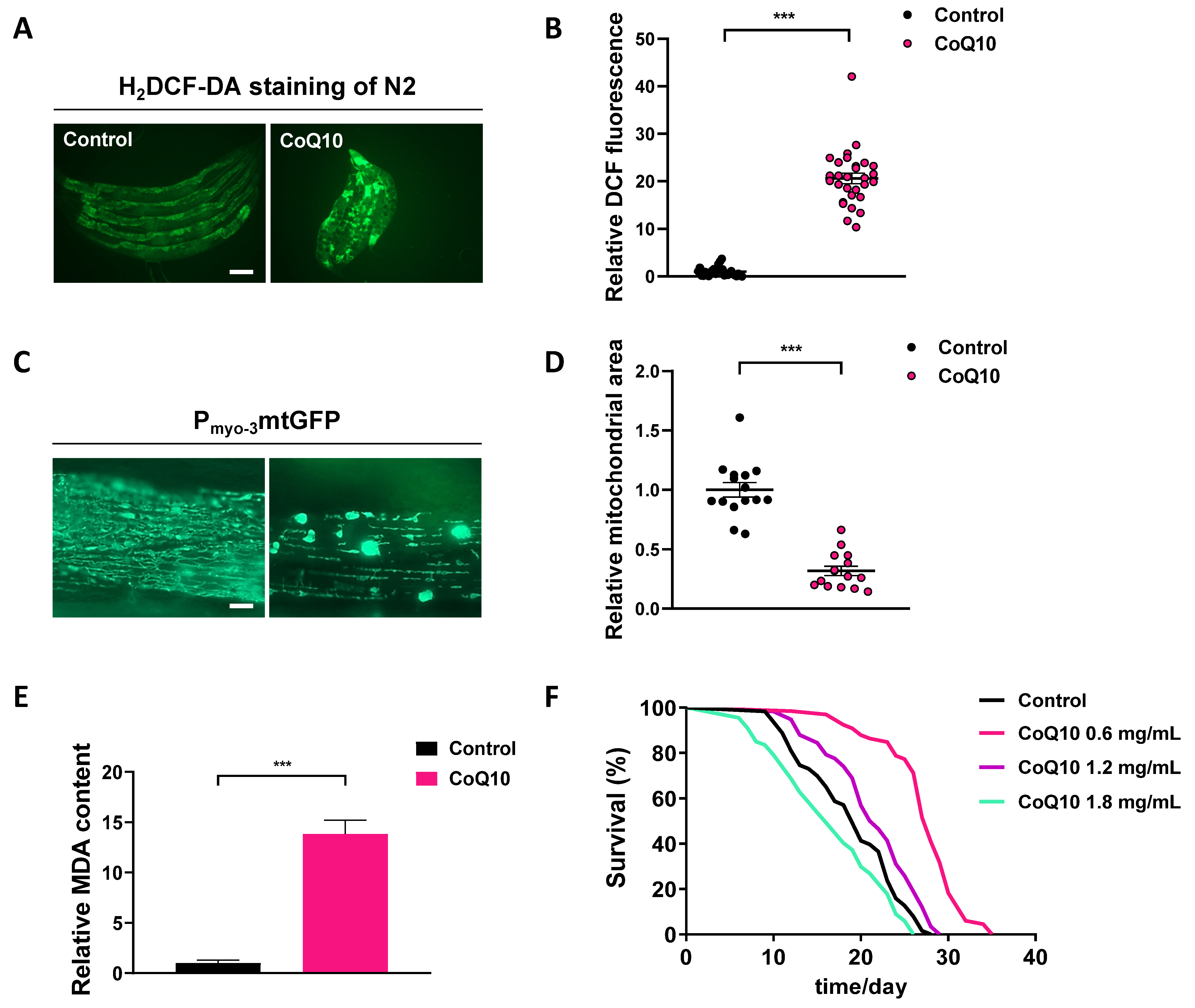

Supplement: Supplemental Information 8 — One-day-old worms were transferred to NGM–OP50 plates or NGM–OP50 plates containing 1.8 mg/mL CoQ10. Physiological endpoints were measured on the day 5. (A) Endogenous ROS was measured through end point microscope DCF fluorescence imaging. Scale bar, 200 µm. (B) Quantified fluorescence intensity of DCF in each group. n = 29. (C) Representative pictures of mitochondria in the C.elegans transgenic strain SJ4103 carrying a mitochondrial GFP reporter driven by the muscle-specific myo-3 promoter. Scale bar, 10 µm. (D) GFP quantification of the mitochondrial area in the muscle of each group. n = 15. (E) MDA was detected by an assay kit according to the manufacturer’s instruction. (F), Survival curves of N2 worms treated with different concentrations of CoQ10. Control, n = 63; CoQ10 (0.6 mg/mL), n = 66; CoQ10 (1.2 mg/mL), n = 58; CoQ10 (1.8 mg/mL), n = 67. Each experiment was repeated at least twice. ∗∗∗ p < 0.001. [file peerj-10-12866-s008.png]
